# Fully-Automated Segmentation of Nasopharyngeal Carcinoma on Dual-Sequence MRI Using Convolutional Neural Networks

**DOI:** 10.3389/fonc.2020.00166

**Published:** 2020-02-19

**Authors:** Yufeng Ye, Zongyou Cai, Bin Huang, Yan He, Ping Zeng, Guorong Zou, Wei Deng, Hanwei Chen, Bingsheng Huang

**Affiliations:** ^1^Department of Radiology, Panyu Central Hospital, Guangzhou, China; ^2^Medical Imaging Institute of Panyu, Guangzhou, China; ^3^Medical AI Lab, School of Biomedical Engineering, Health Science Center, Shenzhen University, Shenzhen, China; ^4^Shenzhen University General Hospital Clinical Research Center for Neurological Diseases, Shenzhen, China; ^5^Department of Oncology, Panyu Central Hospital, Guangzhou, China; ^6^Cancer Institute of Panyu, Guangzhou, China; ^7^Department of Radiology, Shenzhen University General Hospital, Shenzhen, China

**Keywords:** nasopharyngeal carcinoma, magnetic resonance image, dual-sequence, convolutional neural networks, segmentation

## Abstract

In this study, we proposed an automated method based on convolutional neural network (CNN) for nasopharyngeal carcinoma (NPC) segmentation on dual-sequence magnetic resonance imaging (MRI). T1-weighted (T1W) and T2-weighted (T2W) MRI images were collected from 44 NPC patients. We developed a dense connectivity embedding U-net (DEU) and trained the network based on the two-dimensional dual-sequence MRI images in the training dataset and applied post-processing to remove the false positive results. In order to justify the effectiveness of dual-sequence MRI images, we performed an experiment with different inputs in eight randomly selected patients. We evaluated DEU's performance by using a 10-fold cross-validation strategy and compared the results with the previous studies. The Dice similarity coefficient (DSC) of the method using only T1W, only T2W and dual-sequence of 10-fold cross-validation as different inputs were 0.620 ± 0.0642, 0.642 ± 0.118 and 0.721 ± 0.036, respectively. The median DSC in 10-fold cross-validation experiment with DEU was 0.735. The average DSC of seven external subjects was 0.87. To summarize, we successfully proposed and verified a fully automatic NPC segmentation method based on DEU and dual-sequence MRI images with accurate and stable performance. If further verified, our proposed method would be of use in clinical practice of NPC.

## Introduction

Nasopharyngeal carcinoma (NPC) is a cancer type arising from the nasopharynx epithelium with a unique pattern of geographical distribution, with high incidence in Southeast Asia and North Africa (1). NPC has an incidence rate of 0.2‰ in endemic regions. Radiation therapy (RT) has come as the only curative treatment because of the anatomic constraints and its sensitivity to irradiation (2).

The accurate delineation of NPC greatly influences radiotherapy planning. NPC cannot be clearly identified from the adjacent soft tissue on computed tomography (CT) image (3). Compared with CT, magnetic resonance imaging (MRI) has demonstrated superior soft tissue contrast, thus has been used as a preferred modality to evaluate the regional, local and intracranial infiltration of NPC. Moreover, NPC has complex anatomical structure and often shares the similar intensities with the nearby tissues. The NPC often present high shape variability, making the NPC segmentation very challenging (4). In clinical practice, NPC are delineated manually by radiologists or oncologists, which is time-consuming and subjective. Compared with manual delineation, automatic segmentation methods can be faster and relatively objective.

The automatic or semi-automatic segmentation methods, such as the traditional machine learning (ML) methods (5–11), have already been applied to NPC segmentation. The traditional ML methods are subjective to extract hand-crafted features with specific methods. Alternatively, convolutional neural networks (CNNs) allow automatic features extraction and have shown great performance in the field of medical image analysis. For NPC segmentation, there are some studies based on CNNs. Wang et al. (4) and Ma et al. (12) extracted patches in 2-dimension (2D) MRI images and trained a CNN model to classify the patches for NPC segmentation. Wang et al. (4) showed that CNN performed better in segmentation tasks than the traditional ML methods. However, segmentation by patches would cost much time in training while just makes use of the information of small local regions. To overcome these limitations, other studies have applied fully convolutional network (FCN) (13) or U-net (14) structure in NPC segmentation. Men et al. (15) and Li et al. (16) applied an improved U-net to segment NPC in an end-to-end manner. The fully convolutional structure of U-net allows the network to realize pixel-wise segmentation and to input the whole image for NPC segmentation without extracting patches. Compared with extracting patches on images, fully convolutional structure can segment NPC with global image information and increase the segmentation efficiency. Based on FCN and U-net structure, there have been studies about NPC segmentation on multimodality images. Huang et al. (17) applied an improved U-net to segment NPC in PET and CT images. Ma et al. (18) applied a combined CNN to segment NPC in CT and MRI images. Similar to previous studies, the segmentation performance by using multimodality information was better than those by using single modality information. Recently, Huang et al. (19) proposed a dense convolutional network (DenseNet) showing great performance in the field of computer vision. DenseNet uses a densely connected path to concatenate the input features with the output features, enabling each micro-block to receive raw information from all previous micro-blocks. Inspired by the successful application of deep CNNs in NPC segmentation, in this study we proposed a dense connectivity embedding U-net (DEU) based on U-net, dense connectivity and dual-sequence MRI for accurate and automatic segmentation of NPC.

## Materials and Methods

### Patient Data Acquisition and Pre-processing

Totally 44 NPC patients were retrospectively recruited from ^****^ with 34 males and 10 females. The age of the patients ranged from 34 to 73 years old. The ethics committee of Panyu Central Hospital performed the ethical review and approved this study and waived the necessity to obtain informed written consent from the patients. The T1-weighted (T1W) and T2-weighted (T2W) images were both acquired with a 1.5T Siemens Avanto scanner (Siemens AG Medical Solutions, Erlangen, Germany). The spatial resolution of T1W images are 0.93 × 0.93 × 4 mm^3^ and T2W images are 0.48 × 0.48 × 4 mm^3^. The scanning range was from mandibular angle to suprasellar cistern (25 slices), or from suprasternal fossae to suprasellar cistern (45 slices). The gold standard of NPC boundary (including the primary tumor and the metastatic lymph node) was manually delineated by an experienced radiologist and double-checked by an experienced oncologist on the T2W images with reference to T1W images and saved as the gold standard images with a value of one in the lesions and 0 in the other regions. The diagnostic criteria for a detectable lymph node included the following: (1) lateral retropharyngeal nodes with a minimal axial dimension of ≥5 mm and 10 mm for all other cervical nodes, except for the retropharyngeal group, and if the minimum axial dimension of the lymph nodes ≥6 was considered high-risk metastatic; (2) lymph nodes with a contrast-enhancing rim or central necrosis; (3) nodal grouping (i.e., the presence of three or more contiguous and confluent lymph nodes as clusters); (4) extracapsular involvement of lymph nodes.

To make use of the information of both T1W and T2W images, we performed co-registration of T1W to T2W images by using Mattes mutual information (20) as correlation metric. To do this, a one plus one evolutionary method (21) (initial radius 0.004, maximum iterations 300) was used to find the best parameters. To resample the T2W images to the same spatial resolution as T1W images, the T2W images and the gold standard images of the same patients were down-sampled by using linear interpolation. The length and width of the T2W images and gold standard images were reduced by 50%. All the T2W and T1W images were normalized by performing min-max normalization. All the image slices were padded zero and cropped into 256 × 256 dimension. Totally 1950 pairs of T1W and T2W images were used for this study.

### Automatic Segmentation of NPC by Deep Learning

To study the advantage of integrating dual-sequence information, we designed an experiment to compare different inputs, by using 10-fold cross-validation strategy. We trained three models for the comparisons of different inputs, namely, using only T1W, using only T2W and dual-sequence (both T1W and T2W) MRI images, respectively.

#### Comparison Between Different Inputs

##### Network architecture

**Figure 1:**
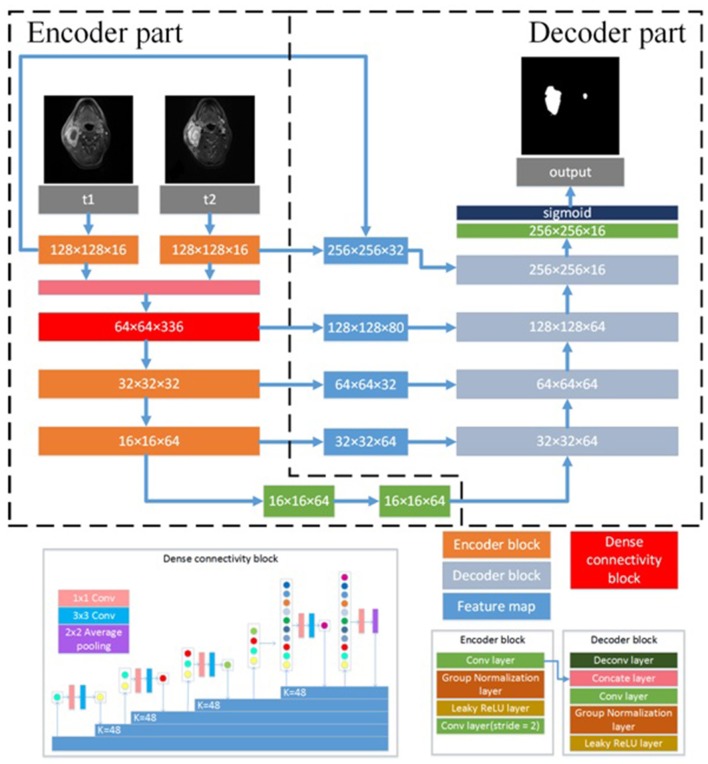
Architecture of the proposed CNN model. N × N × C, N is the size of feature map and C is the number of feature maps. N × N Conv, the convolutional layer with N × N kernel size; K = N, N is the growth filters number; N × N Average pooling, the average pooling layer with N × N kernel size; Concate layer, the concatenation layer; ReLU, rectified linear unit.

We developed a DEU to inherit both advantages of dense connectivity and U-net-like connections. The network architecture is shown in Figure 1. The T1W and T2W images were inputted into the network by two independent paths, respectively. As an end-to-end segmentation framework, the structure consists of an encoder part and a symmetric decoder part.

The encoder part reduces the size of input data sets and extracts high representative features effectively. The decoder part recovers the extracted features to the same size of input images by deploying deconvolution, which is transposed convolution for upsampling. The encoder part consists of four encoder blocks and a dense connectivity block. An encoder block contains three 3 × 3 convolutional (conv) layers, two group normalization (GN) (22) layers and two leaky rectified linear unit (LReLU) (23) layers. The outputs of convolutional layer are inputted into a GN layer, and the groups of GN layer were set as 8. Because GN has shown better performance than batch normalization (BN) (24) with small batches (22), we employed GN in the proposed network. To optimize the effect of training and prevent gradient vanishing or exploding, each convolutional layer is followed by LReLU to the output of GN layer. The convolutional layer with two strides is designed for downsampling the feature maps. LReLU is defined as:

(1) $$\begin{array}{l} \;{\text{y}}_{\text{i}}\;=\;\{\begin{array}{c} {\text{x}}_{\text{i }}\;,\;{\text{x}}_{\text{i}}\geq 0\\ {\alpha \text{x}}_{\text{i}}\;,\;{\text{x}}_{\text{i}}<0 \end{array} \end{array}$$

where α between 0 and 1 decides the slope of the negative part. It is set as 0.1 in the proposed network.

The dense connectivity block consists of a dense block and a transition block. The dense block is a direct connection from any layer to all subsequent layers, motivated by bottleneck structure (25). The dense block consists of GN-LReLU-conv (1 × 1 kernel size)-GN-LReLU-conv (3 × 3 kernel size). The transition block consists of a GN layer and an 1 × 1 convolutional layer followed by a 2 × 2 average pooling layer. Two encoder blocks are used to extract the low-level features of T1W images and T2W images, respectively, and then concatenate them in channel-wise as the input of the dense connectivity block. Another two encoder blocks are designed for the permutation and combination of the low-level features from the output of the dense connectivity block to acquire high-level features.

The decoder part consists of five decoder blocks. A decoder block contains a 3 × 3 deconvolutional layer (deconv), a concatenation layer, two 3 × 3 convolutional layers with stride 2, two LReLU and two GN layers. Deconvolution may cause information loss of the high-resolution images. To address this problem, the concatenation layer is used to fuse the feature maps in the convolution layers from the encoder part with the current feature maps in the deconvolutional layer. These skip-layers are able to capture more multi-scale contextual information and improve the accuracy of segmentation. At the final layer the feature maps are computed by a 1 × 1 convolutional layer with pixel-wise sigmoid.

With all the decoder blocks, the decoder part finally reconstructs the feature maps to an output image with the size of 256 × 256, the same as that of the input images. For the network optimization, the Dice loss (26) between the gold standard and the segmented results is calculated as objective function.

##### Model implementation details

We implemented the proposed DEU in Keras (27) using Tensorflow (28) backend, and trained it on Nvidia Geforce GTX 1080 TI with 11 GB GPU memory. The batch size was set as 1. We used Adam (29) optimizer with a learning rate of 0.0001 and the epochs number of 200. During each training epoch, data augmentation was applied to enlarge the training dataset and to reduce overfitting by flipping and re-scaling each image.

To further improve the segmentation accuracy, we performed post-processing to refine the segmentation results. Since the 2D network may ignore the context information of neighboring slices, the segmentation results with 2D network may include some isolated false positive (FP) areas. We extracted the segmentation results by using connected components algorithm on 3D images for each patient. We then removed the isolated regions which were segmented in only one slice to improve the segmentation accuracy.

The network architecture for the single sequence model is different from the dual-sequence model which is shown in Figure 1. The single sequence model has one single path for extracting features at the beginning of the network. The outputs of the first encoder block are fed to the dense connectivity block directly. The other structure of the single sequence model is the same as in DEU. We evaluated the single sequence model by using 10-fold cross-validation strategy and compared the performance of single sequence models with the dual-sequence model by using Mann-Whitney *U* test. We collected seven additional cases as an external validation dataset to evaluate the robustness and generalization ability of our dual-sequence model.

##### Performance evaluation

We used the testing dataset to evaluate the segmentation performance of all models by calculating Dice similarity coefficient (DSC) (30), sensitivity and precision as follows:

(2) $$\begin{array}{l} \text{DSC}\;=\;\frac{2\text{TP}}{\text{FP}+2\text{TP}+\text{FN}} \end{array}$$

(3) $$\begin{array}{l} \text{Sensitivity}\;=\;\frac{\text{TP}}{\text{TP}+\text{FN}} \end{array}$$

(4) $$\begin{array}{l} \text{Precision}\;=\;\frac{\text{TP}}{\text{TP}+\text{FP}} \end{array}$$

where true positive (TP) denotes the correctly identified tumor area, FP denotes the normal tissue that was incorrectly identified as a tumor, false negative (FN) denotes the tumor area that is incorrectly predicted as normal tissue. DSC describes the overlap between the segmentation results and the gold standard of NPC. Sensitivity describes the overlap between the correctly identified tumor area and the gold standard of NPC. Precision describes the ratio of the correctly identified tumor area in the segmentation result.

#### Comparison With Previous Studies

We evaluated the proposed method by using 10-fold cross-validation strategy. We also compared our results of DEU with the previous studies. However, performing a direct comparison across different studies is difficult due to differences in the datasets. Therefore, we directly compared our results with those in these publications, in terms of DSC. Although they may not be reasonably comparable, these comparisons to some extent provide insights about how our method outperforms the similar studies.

## Results

### Comparison Between Different Network Inputs

**Figure 2:**
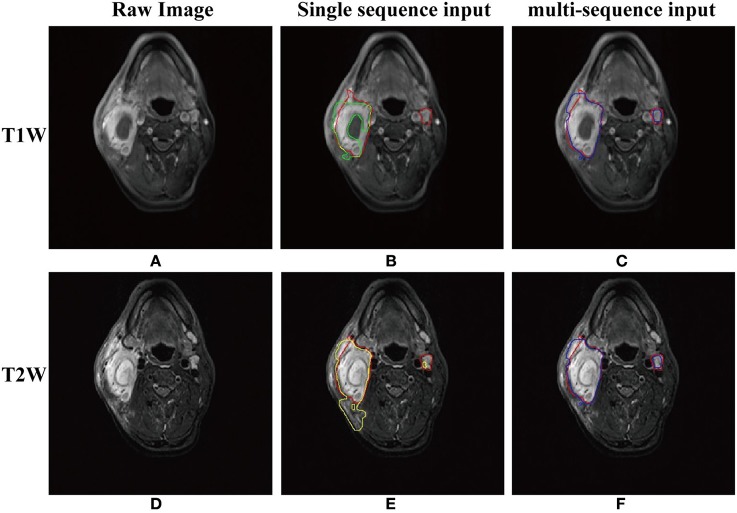
An example of the segmentation results with T1W only, T2W only and dual-sequence images. **(A)** T1W image. **(B)** Automatic segmentation result with T1W image only (green line) and gold standard (red line) presented on the T1W image. Part of the lesion presented lower signal intensity in T1W image (arrow). **(C)**Automatic segmentation result with dual-sequence images (blue line) and gold standard (red line) presented on the T1W image. **(D)** T2W image. **(E)** Automatic segmentation result with T2W only image (yellow line) and gold standard (red line) presented on the T2W image. Some normal tissue beside the tumor presented high signal intensity as compared with the surrounding tissue (arrow). **(F)** Automatic segmentation result with dual-sequence images (blue line) and gold standard (red line) presented on the T2W image.

**Figure 3:**
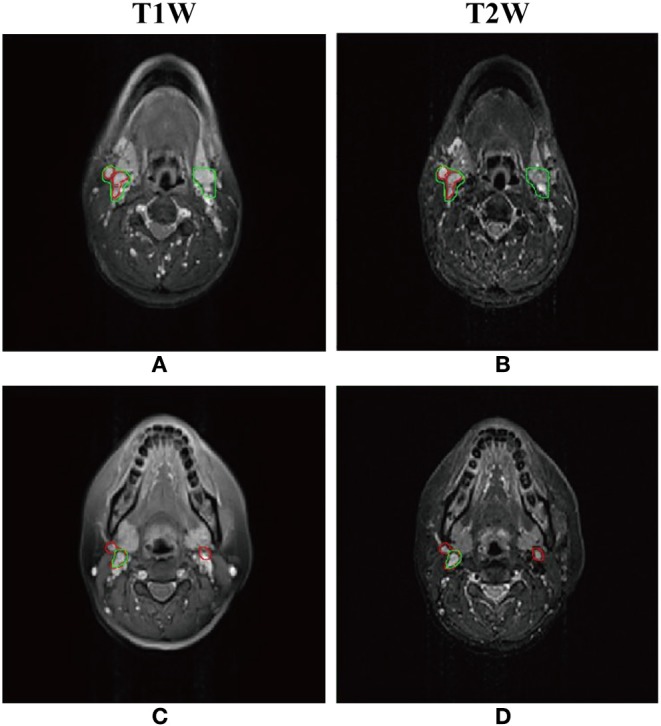
Two typical examples of NPC segmentation with low accuracy. The Dice similarity coefficient (DSC) of first row and second row are 0.610 and 0.467, respectively. **(A,C)** Automatic segmentation result with dual-sequence images (green line) and gold standard (red line) presented on the T1W image. **(B,D)** Automatic segmentation result with dual-sequence images (green line) and gold standard (red line) presented on the T2W image.

As shown in Table 1, the mean DSC, sensitivity, precision of the models with different inputs (T1W only, T2W only, and dual-sequence) were 0.620 ± 0.064, 0.642 ± 0.118 and 0.721 ± 0.036, respectively in 10-fold cross-validation experiment. There were significant differences between the DSC values between the single sequence models and the dual-sequence model (T1W vs. dual-sequence, *p* ≤ 0.01; and T2W vs. dual-sequence, *p* = 0.047), by Mann-Whitney *U* test. The mean DSC with dual-sequence MRI images input was higher than that with single-sequence MRI images input. An example of automatic segmentation result is shown in Figure 2, in which the DSC of our proposed method using only T1W, only T2W and dual-sequence MRI images were 0.721, 0.784, and 0.912, respectively. Two typical examples with poor results are shown in Figure 3, in which the DSC of our proposed method using dual-sequence MRI images were 0.610 and 0.467, respectively. The average DSC of these seven external cases was 0.87.

**Table 1:** Comparisons of segmentation performance between different MRI sequences using 10-fold cross-validation strategy.

**Input**	**DSC^a^**	**Sensitivity**	**Precision**
T1W	0.620±0.064	0.642±0.070	0.654±0.072
T2W	0.642±0.118	0.654±0.115	0.688±0.146
T1W+T2W	0.721±0.036	0.712±0.045	0.768±0.045

a *DSC, Dice similarity coefficient*.

### Comparison With Other Studies

With our trained DEU model in 10-fold cross-validation experiment, a tumor segmentation task for an example (a co-registered T1W image and a T2W image, two-dimensional) took about 0.02 s, and <1 s for a patient. The feature maps of DEU are shown in Figure 1A.

The median DSC in 44 patients was 0.735 (range, 0.383–0.946). The mean DSC, mean sensitivity, mean precision of all patients were 0.721 ± 0.036, 0.712 ± 0.045, 0.768 ± 0.045. The results of previous studies about NPC segmentation in MRI are shown in Table 2. The DSC in the study by Li et al. (16) was 0.736, however in their study they manually selected the images of tumor for segmentation, which means that their method was semi-automatic. Deng et al. (10) and Ma et al. (12) achieved a high DSC of 0.862 and 0.851, respectively, however, their method was applied on the MRI images containing the tumor and was also semi-automatic. Ma et al. (18) obtained mean DSC of 0.746, however, their method was applied on the slices containing the nasopharynx region. Song et al. (8), Yang et al. (9), and Huang et al. (17) obtained mean DSC of 0.761, 0.740 and 0.736, respectively, which was a little higher than our DSC, however in their study PET/CT images were used. The performance of Wang et al. (4) method (mean DSC of 0.725) was very close to our method, which was evaluated in only four patients. Men et al. (15) segmented NPC based on CT images and the mean DSC of 0.716 was slightly lower than ours.

**Table 2:** Comparisons of segmentation performance between our proposed CNN model and the similar studies.

**Studies**	**Algorithm**	**Images used**	**Average DSC^a^**	**Patient number**	**Journal**
Deng et al. (10)	SVM^b^	DCE-MRI^c^	0.862	120	Contrast Media and Molecular Imaging, 2018
Song et al. (8)	Graph-based cosegmentation	PET	0.761	2	IEEE Transactions on Medical Imaging, 2013
Yang et al. (9)	MRFs^d^	PET, CT, MRI	0.740	22	Medical Physics, 2015
Stefano et al. (11)	AK-RW^e^	PET	0.848	18	Medical and Biological Engineering and Computing, 2017
Wang et al. (4)	CNN^f^	MRI	0.725	15	Neural Processing Letters, 2018
Ma et al. (12)	CNNs+3D graph cut	MRI	0.851	30	Experimental and Therapeutic Medicine, 2018
Men et al. (15)	DDNN^g^	CT	0.716	230	Frontiers in Oncology, 2017
Li et al. (16)	CNN	CE-MRI	0.890	29	Biomed Research International, 2018
Huang et al. (17)	CNN	PET-CT	0.736	22	Contrast Media and Molecular Imaging, 2018
Ma et al. (18)	C-CNN^h^	CT-MRI	0.746	90	Physics in Medicine and Biology, 2019
Proposed method	CNN	Dual-sequence MRI	0.721	44	–

a DSC, Dice similarity coefficient;

b SVM, support vector machine;

c DCE-MRI, dynamic contrast-enhanced magnetic resonance imaging;

d MRFs, Markov random fields;

e AK-RW, adaptive random walker with k-means;

f CNN, convolutional neural network;

g DDNN, deep deconvolutional neural network;

h *C-CNN, combined convolutional neural network*.

## Discussion

We proposed an automated NPC segmentation method based on dual-sequence MRI images and CNN. We achieved better performance with dual-sequence MRI images than with single-sequence MRI images, as shown in Table 1 and Figure 2. The good performance on the external validation dataset indicated that our model was robust.

As shown in Figures 2A,D, the image features of NPC in the T1W image and the T2W image were different. The T1W image depicted part of the lesion as a region with lower signal intensity (Figure 2B, arrow). Such low signal intensity region was incorrectly identified as normal tissue, because the network could not gain tumor features from the low signal intensity region. As shown in Figure 2D, the boundary of NPC was clearly shown in T2W image which was easy to segment by the network. Some normal tissue beside the tumor (Figure 2E, arrow) presented high signal intensity as compared with the surrounding tissue. This may cause that the normal tissue beside the tumor was incorrectly identified as tumor (Figure 2E). The proposed method extracted the different features from T1W and T2W images by two independent paths and fused them in the dense connectivity block. As show in Figures 2C,F, the high accuracy result with dual-sequence MRI showed that the different image information was fused as efficient features for more accurate segmentation.

The proposed CNN model has shown advantages in feature extraction and feature analysis. As shown in Figure A1, the feature maps of encoder part might have relatively high spatial resolution, but the features of tumor were not emphasized, since the encoder part was designed for extracting the features of tumor and normal tissue. The decoder part reconstructed the feature maps from encoder part to output the segmentation results, and in this procedure, the features of tumor were emphasized. As shown in Figure A1, the tumor in the feature map of the decoder part showed high signal intensity but had low spatial resolution. The skip-layer structure fused the high spatial resolution feature maps from encoder part and the feature maps of decoder part. The tumor in fused feature map showed high spatial resolution and high signal intensity. The feature maps showed that the skip-layer improved the accuracy of segmentation. To summarize, our proposed network showed accurate NPC segmentation in dual-sequence MRI images.

As shown in Table 2, the mean DSC of the proposed method in 10-fold cross-validation experiment was 0.721 and some studies have reported higher DSC than ours. However, in the studies by Deng et al. (10), Stefano et al. (11), Song et al. (8), Ma et al. (12, 18), and Li et al. (16), their methods were not fully automatic with which the tumor were segmented in the manually drawn volume of interest. The proposed method allowed fully automatic tumor segmentation. Yang et al. (9) and Huang et al. (17) used tumor metabolic information in PET images which made the tumor was easily to be detected. Wang et al. (4) evaluated their method with only four patients. To summarize, we proposed a fully automatic segmentation method with accurate and stable performance in dual-sequence MRI images.

Our proposed method has some limitations. Firstly, the patients sample size was relatively small, and the patients were collected from single center. Future work with a lager sample, especially from multicenter, would be necessary to further verify our method. Secondly, the segmentation performance of the proposed method was unsatisfactory in some small lymph nodes. As shown in Figure 3, part of the normal lymph node and tumor were incorrectly identified. The reason may be that the image feature of normal lymph nodes was similar to that of abnormal lymph nodes in the axial MRI images. We may adapt the DEU to multi-view MRI images in future work. Thirdly, the co-registration of T1W and T2W images is still challenging. A method without co-registration may be proposed in future work.

In this study, we successfully proposed and verified an accurate and efficient automatic NPC segmentation method based on DEU and dual-sequence MRI images. Although both DenseNet and UNet has been applied widely in tumor segmentation tasks, no article has been published combining them for the automatic segmentation of the NPC on dual-sequence MRI. We the first time applied this method in the automatic segmentation of the NPC and showed more stable and better performance than other methods. With dual-sequence images, the combination of different features from T1W and T2W images increased the segmentation accuracy. The DEU extracted the features of T1W and T2W in different path automatically and fused the features with dense connectivity block, which also contributed to the increased accuracy. 10-fold cross-validation results showed that the proposed method gained good performance. Future studies may aim to improve the segmentation accuracy with improved network structure or domain knowledge, avoiding the co-registration between different modalities. If further verified with lager sample and multicenter data, our proposed method would be of use in clinical practice of NPC.

## Data Availability Statement

The datasets for this article are not publicly available because Panyu Central Hospital, the center from which the data were collected, does not agree to make the data publicly accessible. Requests to access the datasets should be directed to Prof. Bingsheng Huang, huangb@szu.edu.cn.

## Ethics Statement

44 NPC patients were retrospectively recruited from Panyu Central Hospital. The ethics committee of Panyu Central Hospital performed the ethical review and approved this study, and waived the necessity to obtain informed written consent from the patients.

## Author Contributions

ZC, HC, BingH, and YY contributed conception and design of the study. YY, HC, YH, WD, and GZ organized the database. ZC and BinH performed the statistical analysis. ZC, BinH, and PZ wrote the first draft of the manuscript. All authors contributed to manuscript revision, read and approved the submitted version.

### Conflict of Interest

The authors declare that the research was conducted in the absence of any commercial or financial relationships that could be construed as a potential conflict of interest.
