# Supplementary material for: Fully-Automated Segmentation of Nasopharyngeal Carcinoma on Dual-Sequence MRI Using Convolutional Neural Networks
Source: Front Oncol. 2020 Feb 19;10:166. doi: 10.3389/fonc.2020.00166 (PMC7045897; doi:10.3389/fonc.2020.00166)
Supplement: Supplementary file 1 [file Image_1.pdf]

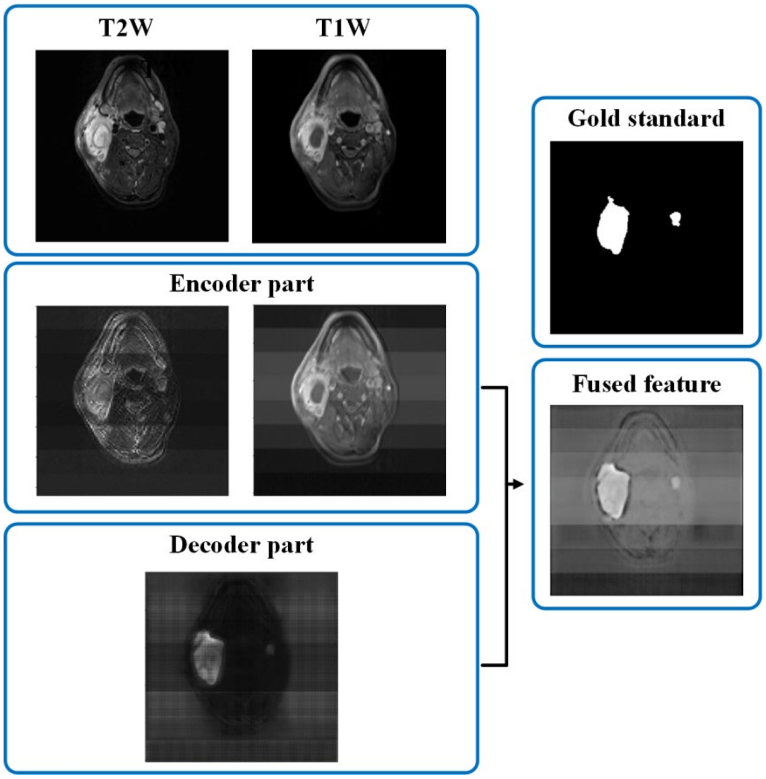

Figure A1. The feature maps of DEU. Both T2W and T1W images are the input of network. The feature maps of "encoder part" are the output from the first encoder block. The feature map of "decoder part" is the output of the last decoder part. The feature map of "fused feature" show the feature after fusing encoder part and decoder part features.
